# Crossed Connections From Insular Cortex to the Contralateral Thalamus

**DOI:** 10.3389/fncir.2021.710925

**Published:** 2021-12-07

**Authors:** Tolulope Adeyelu, Tanya Gandhi, Charles C. Lee

**Affiliations:** Department of Comparative Biomedical Sciences, Louisiana State University, School of Veterinary Medicine, Baton Rouge, LA, United States

**Keywords:** thalamus, insular cortex (IC), corticothalamic circuitry, VPMpc, contralateral connections, chemosensation, nociception, interoception

## Abstract

Sensory information in all modalities, except olfaction, is processed at the level of the thalamus before subsequent transmission to the cerebral cortex. This incoming sensory stream is refined and modulated in the thalamus by numerous descending corticothalamic projections originating in layer 6 that ultimately alter the sensitivity and selectivity for sensory features. In general, these sensory thalamo-cortico-thalamic loops are considered strictly unilateral, i.e., no contralateral crosstalk between cortex and thalamus. However, in contrast to this canonical view, we characterize here a prominent contralateral corticothalamic projection originating in the insular cortex, utilizing both retrograde tracing and cre-lox mediated viral anterograde tracing strategies with the Ntsr1-Cre transgenic mouse line. From our studies, we find that the insular contralateral corticothalamic projection originates from a separate population of layer 6 neurons than the ipsilateral corticothalamic projection. Furthermore, the contralateral projection targets a topographically distinct subregion of the thalamus than the ipsilateral projection. These findings suggest a unique bilateral mechanism for the top-down refinement of ascending sensory information.

## Introduction

The canonical picture of sensory information processing depicts a serial flow of information ascending from the periphery, eventually reaching the thalamus and then the cerebral cortex (Felleman and Van Essen, 1991; Rouiller et al., 1991; Bullier and Nowak, 1995; Carrasco and Lomber, 2009). The thalamus is also the target of numerous descending projections originating from cortical layer 6 that refine and modulate this incoming sensory stream (Reichova and Sherman, 2004; Briggs and Usrey, 2009; Thomson, 2010; Lee et al., 2012; Olsen et al., 2012; Augustinaite et al., 2014). In addition, a separate corticothalamic pathway originating from layer 5 has been implicated in the feedforward processing of information to higher thalamic nuclei, which in turn project to higher cortical areas (Sherman and Guillery, 2002; Reichova and Sherman, 2004; Lee and Sherman, 2008; Llano and Sherman, 2008; Lee, 2015). These interactions between thalamic nuclei and cortical areas are generally viewed as being restricted unilaterally, with interhemispheric communication not considered as occurring between cortex and thalamus (Jones, 1981, 2001; Ghosh et al., 1990; Grant et al., 2012; Brandt and Dietrich, 2019). Interestingly though, this hemispheric segregation of thalamus from cortex is not globally true, with several bilaterally crossed connections identified between midline thalamic nuclei and cortical areas (Reep and Winans, 1982; Molinari et al., 1985; Preuss and Goldman-Rakic, 1987; Dermon and Barbas, 1994; Carretta et al., 1996; Négyessy and Bentivoglio, 1998; Alloway et al., 2008). These crossed connections have been mainly implicated in higher-order cognitive processes, such as memory, but some may be involved in the processing of sensory information (Reep and Winans, 1982; Preuss and Goldman-Rakic, 1987; Dermon and Barbas, 1994; Carretta et al., 1996; Négyessy and Bentivoglio, 1998).

The insular cortex is a key site for the processing of sensory information from several modalities (nociception, chemosensation, interception, etc.) and is the target of multiple convergent inputs, including ipsi- and contra-lateral cortical, limbic, neuromodulatory, as well as ascending thalamic inputs from the parvicellular part of the ventral posteromedial nucleus (VPMpc; Kosar et al., 1986b; Cechetto and Saper, 1987; Ogawa et al., 1992; Shi and Cassell, 1998; Nakashima et al., 2000; Schier and Spector, 2019; Gehrlach et al., 2020). This convergence likely results in the complex spatial coding that integrates information across the insular cortical surface (Kosar et al., 1986a; Ogawa et al., 1992; Chen et al., 2011; Nieuwenhuys, 2012; Gogolla et al., 2014; Peng et al., 2015; Fletcher et al., 2017; Gehrlach et al., 2020). Like other cortical areas, layer 6 insular cortical neurons target the ipsilateral thalamus (Shi and Cassell, 1998; Nakashima et al., 2000; Holtz et al., 2015; Schier and Spector, 2019), but intriguingly may also send projections to the contralateral thalamus (Reep and Winans, 1982; Oh et al., 2014).

These potential crossed insular corticothalamic projections from layer 6 have received little attention in prior studies (Shi and Cassell, 1998; Nakashima et al., 2000; Holtz et al., 2015); as such, key questions remain regarding their organization. Among these, do distinct neuronal sources in the cortex target the ipsilateral and contralateral thalamus? And do bilateral corticothalamic projections converge on the same thalamic targets? To address these questions, we employed both retrograde and anterograde tracing approaches to examine these crossed corticothalamic projections in a mouse model system. We employed the Ntsr1-Cre transgenic mouse strain, which expresses Cre-recombinase in a subset of layer 6 cortical neurons that project to the thalamus (Lee et al., 2012; Olsen et al., 2012; Mease et al., 2014; Sundberg et al., 2018; Williamson and Polley, 2019; Augustinaite and Kuhn, 2020a,b; Clayton et al., 2021). Overall, we find a novel crossed corticothalamic projection originating from the insular cortex, whose organization is discussed below.

## Methods

### Animals and Surgery

Adult mice were used to examine the organization of the insular corticothalamic projections. The following procedures were approved by the Institutional Animal Care and Use Committee (IACUC) of the Louisiana State University School of Veterinary Medicine. Wild-type C57BL/6J mice (*n* = 5; strain: 000664; Jackson Labs, Bar Harbor, ME, United States) were used for retrograde tracer injections. Ntsr1 Cre-transgenic mice [Tg(Ntsr1-cre)GN220Gsat; MMRRC, U.C. Davis, Davis, CA, United States] were used to examine the anterograde projections of layer 6 corticothalamic neurons (*n* = 6) and to colocalize retrogradely labeled corticothalamic neurons with Cre-recombinase expressing neurons (*n* = 3). For surgeries, mice were first anesthetized with an injection of a ketamine/xylazine cocktail (at 30 mg/kg), until fully sedated as assessed by toe-pinch withdrawal reflex. The head of the animals were shaved, then secured in a stereotaxic apparatus. The scalp was cleaned with alcohol and betadine, an incision made across the midline, and a craniotomy performed above the injection sites. Following the injections described below, the site was sutured, and a generic triple antibiotic ointment applied. Animals were recovered and monitored daily for any signs of distress following surgery until sacrifice.

### Tracer Injections

To examine the anterograde terminations of layer 6 corticothalamic neurons, we utilized dual injections of “floxed” viruses (800 nl at 200 nl/min) in the insular cortex of Ntsr1-Cre transgenic mice. Viral injections to express either EYFP or mCherry in layer 6 corticothalamic axons [AAV5-CaMKIIa-hChR2(H134R)-EYFP or AAV5-CaMKIIa-eNpHR3.0-mCherry; UNC Vector Core, Chapel Hill, NC, United States] were stereotaxically targeted with a Hamilton Neuros syringe (Hamilton Company, Reno, NV, United States) bilaterally to either the left or right insular cortices, respectively (relative to bregma: AP + 1.54 mm; ML ± 2.7 mm; V −3.5 mm). The animals were euthanized after 21 days to allow for adequate expression of the virally encoded fluorophores. Next, to examine the origins of insular cortical projections to the thalamus, we employed a dual retrograde tracing strategy. Injections (500 nl at 200 nl/min) of fluorogold (Fluorochrome, Denver, CO, United States) and fluororuby (ThermoFisher, Waltham, MA, United States) were stereotaxically targeted using a Hamilton Neuros syringe (Hamilton Company) to the left or right VPMpc, respectively [relative to bregma: anterior–posterior (AP) −2.06 mm; lateral (ML) ± 0.5 mm; ventral (V) −4 mm]. Animals were then allowed to recover for 7 days prior to sacrifice to allow for transport. Finally, to assess colocalization of retrogradely labeled neurons with AAV-transfected, Cre-recombinase positive neurons, we combined both retrograde fluorogold (Fluorochrome) injection in the thalamus and AAV5-CaMKIIa-eNpHR3.0-mCherry (UNC Chapel Hill) in the cortex on the same side of the brain, using the same injection parameters detailed above. These animals were allowed to recover for 21 days to allow for the transport of both retrograde tracer and the expression of the virally encoded fluorophore.

### Histological Analysis

Mice were sacrificed by first anesthetizing with isoflurane inhalation, then transcardially perfusing with 4% paraformaldehyde (PFA) in 10 mM phosphate buffered saline (PBS). The brains were extracted and post-fixed in 4% PFA in 10 mM PBS overnight, followed by transferring to a solution of 30% sucrose/4% PFA in 10 mM PBS for cryoprotection for 3 days. Brains were then blocked in the coronal plane and sectioned at 50 μm thickness with a cryostat (Leica Biosystems, Wetzlar, Germany). A 1:4 series of sections was mounted on gelatinized slides, then coverslipped with anti-fade medium (Vector Labs, Burlingame, CA, United States). To assess colocalization of retrograde labeling with Ntsr1 Cre-recombinase positive neurons in the insular cortex, some sections were stained immunohistochemically with a mouse monoclonal primary antibody (1:1000) for Cre-Recombinase (MAB3120, Clone 2D8, Millipore Sigma, Burlington, MA, United States) and an Alexa488-goat-anti-mouse secondary antibody (1:1000) (A-11001, ThermoFisher Scientific, Waltham, MA, United States), using methods previously described (Sultana et al., 2021).

Sections were imaged using either a Nanozoomer slide scanner (Hamamatsu Photonics, Hamamatsu City, Shizuoka, Japan) or a Nikon-NiU florescence microscope (Nikon Instruments, Tokyo Japan). Nanozoomer captured images were first processed and analyzed using the NDP.view2 software (Hamamatsu Photonics). Nikon-NiU images were captured as Z-stacks, then converted into 2-D images through the extended depth of focus provided by the Nikon Element Advanced Research software (Nikon Instruments). Images were then further analyzed in ImageJ (NIH, Bethesda, MD, United States) to assess counts of retrogradely labeled neurons and fluorescence intensity (Collins, 2007; Grishagin, 2015; Schindelin et al., 2015). Areal and nuclear boundaries were gauged using cyto- and myeloarchitecture discerned from background staining and from gross section morphology compared to a standard atlas (Franklin and Paxinos, 2001). Quantitative data was compiled and analyzed in Excel (Microsoft Corporation, Redmond, WA, United States) and figures were composed in CorelDraw (Corel Corporation, Ottowa, ON, Canada).

## Results

**FIGURE 1:**
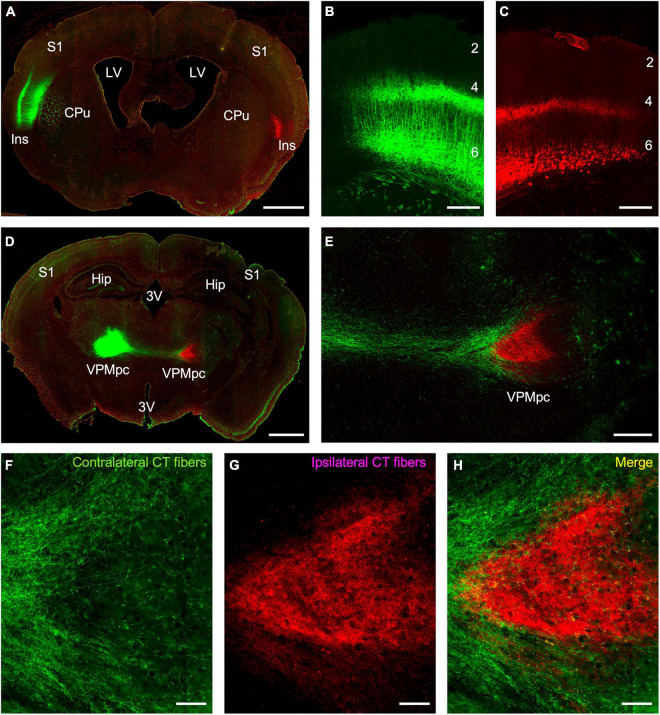
Anterograde tracing of insular corticothalamic projections originating from layer 6 neurons in Ntsr1 Cre-recombinase expressing transgenic mice. **(A)** Injections of “floxed” AAV vectors expressing either EYFP (green) or mCherry (red) were stereotactically targeted to the left and right insular cortices, respectively. **(B,C)** Expression of virally encoded fluorescent proteins labeled the predicted population of layer 6 corticothalamic pyramidal neurons and their dendritic and axonal processes. **(D)** Anterograde fibers from both injections formed dense terminals in the respective ipsilateral thalamus, but also sent fibers to the contralateral thalamus. **(E)** Contralateral fibers (green fibers in this instance) crossed the midline and terminated in a region surrounding the ipsilateral fiber terminations (red fibers in this instance). **(F–H)** Despite the gross separation of terminal fields, some of the ipsilateral and contralateral corticothalamic (CT) labeling formed puncta in closely apposed regions, suggesting possible convergence of bilateral CT inputs on the same thalamic neuron. Abbreviations: CPu: caudate putamen; Hip: hippocampus; Ins: insular cortex; LV: lateral ventricle; S1: primary somatosensory cortex; VPMpc: parvicellular part of the ventral posteromedial nucleus; 3V: third ventricle; #s: layers 1–6. Scale bars: **(A,D)**: 1 mm; **(B–C,E)**: 250 μm; **(F–H)**: 50 μm.

To specify the origins and terminations of the insular corticothalamic projections, we employed a cell-type specific anterograde tracing approach utilizing Ntsr1 Cre-recombinase expressing transgenic mice. We assessed whether the genetically tagged layer 6 neurons in the insular cortex project to both the ipsilateral and contralateral thalamus and whether their terminations overlapped. Dual injections of “floxed” viral constructs expressing either EYFP or mCherry in layer 6 neurons of the left and right insular cortices, respectively, were made and the pattern of anterograde terminations assessed in the thalamus (Figure 1). Viral expression of both fluorophores fully labeled the predicted layer 6 pyramidal neurons and their processes in both insular cortical hemispheres (Figures 1A–C). Expression of fluorophores spanned a broad area (averaging ∼3 mm^2^) and likely encompassed several insular subregions (Figures 1A–C). Interestingly, we found that the Ntsr1-cre tagged layer 6 neurons project to both ipsilateral and contralateral thalami; but on a broad scale, the terminal fields were topographically segregated (Figures 1D–H). Notably, the ipsilateral insular corticothalamic projections terminated primarily in a “core” region of the presumed VPMpc (Figures 1D–E,G), while the contralateral projection terminated in “shell” regions on the dorsal and ventral aspects of the ipsilateral terminal field (Figures 1D–F). The “shell” terminal fields potentially included nuclei outside of the presumed VPMpc core, indicating an asymmetric nuclear organization of the contralateral corticothalamic projections (Figures 1D–F). The contralateral projection was also much less intense than the ipsilateral projection (22.7 ± 8.6% of the total fluorescence intensity from both thalami; averaged across all viral injections), concomitant with the relative contributions suggested by the retrograde results. On a finer scale though, these projections were not strictly segregated, with some labeled axons from each projection found overlapping and some puncta in proximity near void regions (presumed cell bodies), suggesting that bilateral projections may converge on individual thalamic neurons (Figure 1H).

**FIGURE 2:**
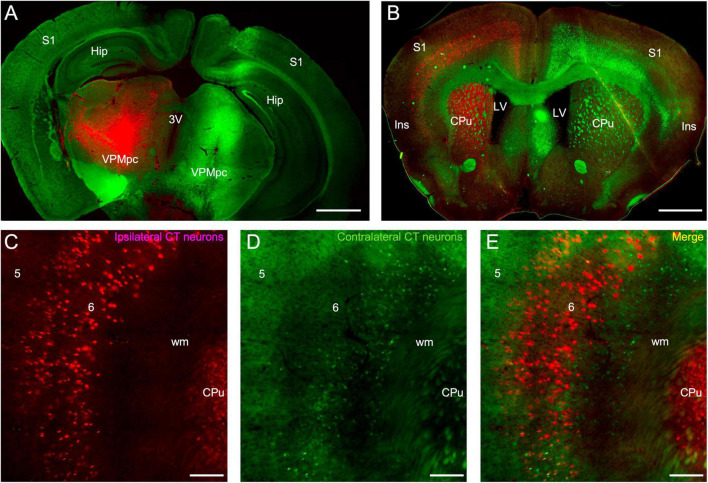
Retrograde labeling of insular corticothalamic neurons. **(A)** Injections of either fluorogold (green) or fluororuby (red) were stereotactically targeted to the left and right VPMpc. Some injection site damage was observed, possibly due to injection speed. **(B)** Retrograde labeling was observed in the respective ipsilateral insular cortical area but was also evident in the contralateral insular cortex. Labeling was also found in other ipsilateral cortical areas, such as S1, indicative of some injection spread into adjacent thalamic nuclei, such as the ventral posterior nuclei (VP) and posteromedial nucleus (POm). **(C–E)** Both the retrogradely labeled ipsilateral CT neurons (red labeling in this instance) and the contralateral CT neurons (green neurons in this instance) originated from layer 6. Contralateral projecting neurons appeared smaller than ipsilateral projecting neurons, perhaps reflecting differences of labeling efficiency or morphological class. However, the contralateral CT neurons were interdigitated with the ipsilateral CT neurons and double-labeled neurons projecting to both thalami were rare, indicating that separate neuronal populations project to either the ipsilateral or contralateral thalami. Abbreviations are the same as in Figure 1. Scale bars: **(A,B)**: 1 mm; **(C–E)**: 200 μm.

The insular origins of corticothalamic projections were then assessed using a dual retrograde tracing approach. Fluorogold was injected into the thalamus of one hemisphere and fluororuby was injected into the other hemisphere (Figure 2). Relatively large thalamic injections were made to ensure near complete labeling of the cortical projection sources (Figure 2A). The pattern of retrograde labeling was then assessed in each cortical hemisphere (Figure 2B). Overall, bilateral corticothalamic projections originated from layer 6 in the insular cortex of both hemispheres, but varied in their relative contribution, with the ipsilateral projection neurons dominating and comprising 87.1 ± 6.9% of the total convergent projection from both insular cortices (Figures 2B–E). Retrogradely labeled neurons were found spanning a broad area in the insular cortex resulting from the large thalamic injections, and likely encompassing several functional thalamic regions (Figures 2B–E). In some cases, retrograde labeling was observed ipsilaterally in surrounding cortical areas, such as S1, and in cortical layer 5, indicative of tracer injection spread into other thalamic nuclei (Figure 2B). Topographically, the ipsilateral and contralateral projection neurons overlapped and originated from similar areal domains across the insular cortical surface (Figures 2B–E). However, double-labeled neurons were exceedingly rare (<1%), indicating that separate populations of layer 6 corticothalamic neurons target either the ipsilateral or contralateral thalamus, with no (or vanishingly few) branched axons connecting both (Figure 2E). Furthermore, the contralateral corticothalamic projection neurons appeared to originate from a lower sublaminar region than the ipsilateral projection (Figures 2C–E).

**FIGURE 3:**
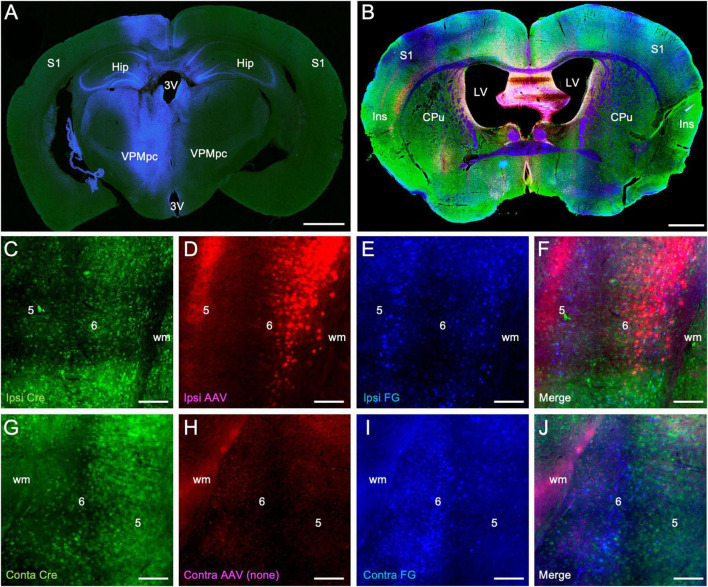
Colocalization of retrograde corticothalamic labeling with Ntsr1-Cre-recombinase positive insular corticothalamic neurons. **(A)** Injections of fluorogold (blue) were stereotactically centered in the left VPMpc, as in Figure 2. **(B)** In the same animal, injection of an AAV vector expressing mCherry (red) was stereotactically targeted to the ipsilateral insular cortex, as in Figure 1. Retrogradely labeled sections were immunohistochemically stained for Cre-recombinase expression (green). **(C–F)** Retrogradely labeled corticothalamic neurons (blue) were again observed in layer 6 of the insular cortex. Many retrogradely labeled neurons (blue) were found to colocalize with both Cre-recombinase positive neurons (green) and with neurons expressing mCherry (red) following viral transfection. **(G–J)** Similarly, in the contralateral insular cortex, retrogradely labeled neurons (blue) were found to colocalize with Cre-recombinase positive neurons (green), although to a lesser extent than that observed in the ipsilateral insular cortex. Abbreviations are the same as in Figure 1. Scale bars: **(A,B)**: 1 mm; **(C–J)**: 100 μm.

Finally, we assessed the extent to which the population of Ntsr1-Cre-recombinase expressing neurons overlapped with retrogradely labeled corticothalamic neurons. In these experiments, fluorogold was injected into the thalamus (blue labeling) and an AAV vector expressing mCherry (red labeling) injected into the insular cortex, both in the left hemisphere (Figures 3A–B). Sections were subsequently stained immunohistochemically for Cre-recombinase expressing neurons (green labeling Figures 3B,C,G). We found significant double-labeling of retrogradely labeled layer 6 corticothalamic neurons in the overlap zone of the ipsilateral insular cortex with Cre-recombinase expressing neurons identified immunohistochemically (blue/green: 83.6 ± 11.9%), virally (blue/red: 66.4 ± 16.7%), and with all combined (blue/red/green: 40.5 ± 13.3%). In the contralateral insular cortex, we also observed double-labeling of retrogradely labeled cells with immunohistochemically identified Cre-recombinase expressing neurons, albeit at a reduced level (blue/green: 29.3 ± 6.6%), which is consonant with the relatively reduced intensity of anterogradely labeled contralateral corticothalamic fibers (Figure 1) and the topographic separation of retrogradely labeled ipsilateral and contralateral corticothalamic neuronal cell bodies (Figure 2).

## Discussion

We examined the origins and terminations of bilateral insular corticothalamic projections using retrograde and cell-type specific anterograde tracing approaches (Figures 1–3). Methodologically, both approaches have their caveats. First, our anterograde tracer studies focused on identifying the projection patterns of a genetically specified neuronal population, i.e., the Ntsr1 Cre-recombinase tagged layer 6 corticothalamic neurons (Lee et al., 2012; Olsen et al., 2012; Mease et al., 2014; Sundberg et al., 2018; Williamson and Polley, 2019; Augustinaite and Kuhn, 2020a,b; Clayton et al., 2021). As expected, we found that this population of layer 6 neurons was a major source of the corticothalamic projection to the ipsilateral thalamus (∼80%), but comprised about a quarter of the contralateral projection (Figure 3). As such, there are likely other neuronal subtypes in the insular cortex that project to the ipsilateral and contralateral thalamus, which remain to be investigated (Reep and Winans, 1982; Holtz et al., 2015; Fletcher et al., 2017; Schier and Spector, 2019).

In addition, in our retrograde studies, we made relatively large thalamic injections to completely label the insular corticothalamic projections. The injection sites often encompassed not only the VPMpc, but surrounding nuclei as well (Figure 2A). As such, the retrogradely labeled insular cortical neurons may represent targets to other thalamic nuclei. From our injection site analyses and based on prior studies, such involvement is possible, since the other main target of insular corticothalamic projections, the mediodorsal nucleus (MD), may have been labeled following some injections. However, corticothalamic projections to these higher-order nuclei are distinguished by their origins in layer 5 and thus were readily distinguished from the feedback corticothalamic projections originating in layer 6. Nevertheless, the overall retrograde results might more precisely be considered as that of the bilateral insular connections to the thalamus, rather than to the VPMpc, *per se*. Future parcelation of these crossed projections topographically and functionally will require more focal injections that specifically target the many insular cortical and related thalamic subregions (Kosar et al., 1986a; Ogawa et al., 1992; Chen et al., 2011; Nieuwenhuys, 2012; Gogolla et al., 2014; Peng et al., 2015; Fletcher et al., 2017; Gehrlach et al., 2020).

**FIGURE 4:**
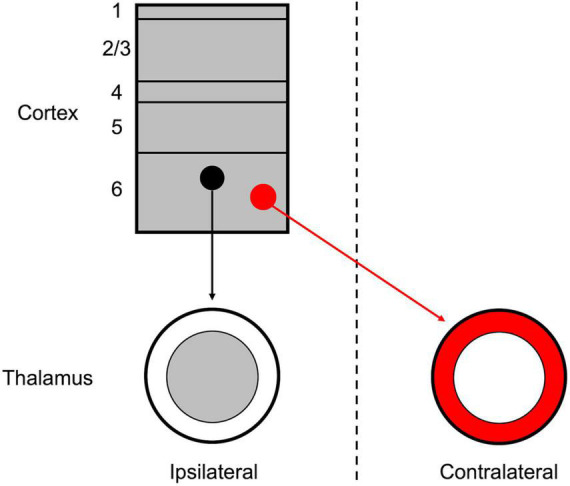
Schematic organization of the layer 6 insular corticothalamic projections. The insular cortex is composed of both ipsilaterally projecting (black) and contralaterally projecting (red) corticothalamic (CT) neurons, which form separate, yet interdigitated, groups across layer 6. The ipsilateral CT projection terminates in a “core” region of the thalamus, the presumed VPMpc, while the contralateral CT projection terminates in a “shell” region surrounding the ipsilateral projection.

Although the thalamus and cortex are typically regarded as unilaterally linked, several exceptions to this “rule” have been noted, primarily among non-sensory and higher-order nuclei (Reep and Winans, 1982; Preuss and Goldman-Rakic, 1987; Dermon and Barbas, 1994; Carretta et al., 1996; Négyessy and Bentivoglio, 1998; Oh et al., 2014). Among these, the insular cortex is a potential source of corticothalamic projections to both the ipsilateral and contralateral thalamus (Reep and Winans, 1982; Oh et al., 2014). In the extensive investigation of mesoscale connectivity in the mouse by Oh et al. (2014), their dataset contains evidence of anterograde labeling of insular cortical projections to both the ipsilateral and contralateral thalami (the presumed VPMpc). In the present study, we have specifically demonstrated that these ipsilateral and contralateral corticothalamic projections originate from separate cortical populations in insular cortical layer 6, which target topographically distinct thalamic regions (Figure 4).

Overall, our findings suggest that layer 6 of the insular cortex is the source of parallel pathways to the ipsilateral and contralateral thalamus. The functional role of the contralateral corticothalamic connections (CCCs) remains to be defined, but in comparison, the layer 6 ipsilateral corticothalamic connections (ICCs) typically are regarded as modulatory pathways (Reichova and Sherman, 2004; Briggs and Usrey, 2009; Thomson, 2010; Lee et al., 2012; Olsen et al., 2012). On a synaptic physiological level, the layer 6 ICCs generally exhibit “modulator” properties, such as a graded-response, paired-pulse facilitation, and activation of both ionotropic and metabotropic glutamate receptors (Bartlett and Smith, 2002; Reichova and Sherman, 2004). Whether the insular layer 6 CCCs also exhibit such “modulator” properties, or alternatively “driver” properties more typical of layer 5 neurons, remains unresolved and could define the potential functional significance of these connections (Molinari et al., 1985; Négyessy and Bentivoglio, 1998; Sherman and Guillery, 2002; Reichova and Sherman, 2004; Alloway et al., 2008; Lee and Sherman, 2008; Llano and Sherman, 2008; Lee, 2015). Moreover, whether and how ICCs and CCCs functionally converge on single thalamic neurons could further denote the role of contralateral descending control of information processing in the thalamus.

The insular cortex is composed of distinct subregions that are implicated in a variety of functions, including integrating sensory information across multiple modalities, e.g., nociceptive, thermoceptive, gustatory, interoceptive, and others (Nieuwenhuys, 2012; Gogolla et al., 2014; Gehrlach et al., 2020). We speculate that the CCCs may engage gustatory and visceroceptive neurons in the VPMpc (Holtz et al., 2015; Fletcher et al., 2017; Schier and Spector, 2019). Given the unique nature of chemosensory information processing, descending cortical control may not necessarily be predicted as restricted hemispherically, and similar crossed connections have been noted for the olfactory thalamus and cortex (Dermon and Barbas, 1994). Considering the nature of the receptor epithelium, lateralization of chemosensation has fewer obvious benefits, compared with other sensory modalities, i.e., vision, audition, and somatosensation (Sherman and Guillery, 2002; Alitto and Usrey, 2003; Briggs and Usrey, 2009). Indeed, integration of bilateral information at the level of the thalamus and cortex may be essential for fully processing incoming chemosensory information. However, the apparent lack of reciprocity of the CCCs, i.e., absent contralateral thalamocortical inputs, remains intriguing (Kosar et al., 1986a; Ogawa et al., 1992; Chen et al., 2011; Nieuwenhuys, 2012; Gogolla et al., 2014; Peng et al., 2015; Fletcher et al., 2017; Gehrlach et al., 2020). At present though, our findings do not resolve whether the observed CCCs are specific to chemosensory processing or more broadly related to other functions noted above. Consequently, future studies are needed to specifically define the role of these crossed corticothalamic projections in normal insular operations.

## Data Availability Statement

The raw data supporting the conclusions of this article will be made available by the authors, without undue reservation.

## Ethics Statement

The animal study was reviewed and approved by the Institutional Animal Care and Use Committee of the Louisiana State University School of Veterinary Medicine.

## Author Contributions

CL: conceived of the study. TA and TG: conducted the experimental procedures. TA, TG, and CL: analyzed the data and drafted and revised the manuscript. All authors contributed to the article and approved the submitted version.

## Conflict of Interest

The authors declare that the research was conducted in the absence of any commercial or financial relationships that could be construed as a potential conflict of interest.

## Publisher’s Note

All claims expressed in this article are solely those of the authors and do not necessarily represent those of their affiliated organizations, or those of the publisher, the editors and the reviewers. Any product that may be evaluated in this article, or claim that may be made by its manufacturer, is not guaranteed or endorsed by the publisher.
